# The Role of Autophagy Modulated by Exercise in Cancer Cachexia

**DOI:** 10.3390/life11080781

**Published:** 2021-08-02

**Authors:** Julia Windi Gunadi, Ariyani Sudhamma Welliangan, Ray Sebastian Soetadji, Diana Krisanti Jasaputra, Ronny Lesmana

**Affiliations:** 1 Department of Physiology, Faculty of Medicine, Maranatha Christian University, Surya Sumantri 65, Jawa Barat 40164, Indonesia; 2 Maranatha Biomedical Research Laboratory, Faculty of Medicine, Maranatha Christian University, Surya Sumantri 65, Jawa Barat 40164, Indonesia; dianakjasaputra67@gmail.com; 3 Undergraduate Program, Faculty of Medicine, Maranatha Christian University, Surya Sumantri 65, Jawa Barat 40164, Indonesia; 1810054@maranatha.ac.id (A.S.W.); 1810095@maranatha.ac.id (R.S.S.); 4 Department of Pharmacology, Faculty of Medicine, Maranatha Christian University, Jawa Barat 40164, Indonesia; 5 Physiology Division, Department of Biomedical Sciences, Faculty of Medicine, Universitas Padjadjaran, Raya Bandung-Sumedang Km 21, Jawa Barat 45363, Indonesia; ronny@unpad.ac.id; 6 Division of Biological Activity, Central Laboratory, Universitas Padjadjaran, Raya Bandung-Sumedang Km 21, Jawa Barat 45363, Indonesia; 7 Center of Excellence in Higher Education for Pharmaceutical Care Innovation, Universitas Padjadjaran, Raya Bandung-Sumedang Km 21, Jawa Barat 45363, Indonesia

**Keywords:** cancer, cachexia, autophagy, combined exercise, aerobic, resistance

## Abstract

Cancer cachexia is a syndrome experienced by many patients with cancer. Exercise can act as an autophagy modulator, and thus holds the potential to be used to treat cancer cachexia. Autophagy imbalance plays an important role in cancer cachexia, and is correlated to skeletal and cardiac muscle atrophy and energy-wasting in the liver. The molecular mechanism of autophagy modulation in different types of exercise has not yet been clearly defined. This review aims to elaborate on the role of exercise in modulating autophagy in cancer cachexia. We evaluated nine studies in the literature and found a potential correlation between the type of exercise and autophagy modulation. Combined exercise or aerobic exercise alone seems more beneficial than resistance exercise alone in cancer cachexia. Looking ahead, determining the physiological role of autophagy modulated by exercise will support the development of a new medical approach for treating cancer cachexia. In addition, the harmonization of the exercise type, intensity, and duration might play a key role in optimizing the autophagy levels to preserve muscle function and regulate energy utilization in the liver.

## 1. Introduction

Patients with cancer usually experience cachexia; cancer cachexia is a multifactorial syndrome correlated with cancer, characterized by skeletal muscle decline that cannot be fully recovered by nutritional support, and eventually leads to dysfunction of the muscle [1,2]. This syndrome manifests in clinical symptoms, such as weight loss, muscle atrophy, anorexia, fatigue, anemia, and edema, which might influence the patient’s quality of life, decrease their sensitivity to treatment, and finally shorten their survival rate [3,4]. According to the international consensus, three criteria can be used to diagnose cancer cachexia: a weight loss > 5% over the past six months, a body mass index < 20 and any degree of weight loss > 2%, or an appendicular skeletal muscle index consistent with sarcopenia and any degree of weight loss > 2%. The consensus also distinguishes three stages of cancer cachexia: precachexia, cachexia, and refractory cachexia [1].

Research shows that the size of cancerous tissue is not associated with the presence of cachexia, but that the stage of cancer can affect the severity of cachexia. The prevalence of cancer cachexia is approximately 50% in all patients with cancer, increasing to 80% according to the disease progression [5]. Almost 80% of patients with gastric or pancreatic cancer and 50% of patients with lung, prostate, or colon cancer experience cachexia. This syndrome also thrives in 40% of patients with breast tumors and some with leukemia [6].

The pathogenesis of cancer cachexia is systemic inflammation caused by the interaction between tumor and organ tissues, resulting in alterations to the metabolism and homeostasis in different parts of the body [5]. Cancer cachexia ultimately affects the skeletal muscle, but its effect is also found in other tissues, such as the cardiac muscle and liver [7]. In the process of cancer cachexia, an imbalance between the synthesis and degradation of protein in the skeletal muscles’ activated signaling pathways potentially provokes muscle atrophy [2]. Autophagy plays an important role in protein degradation, along with two other main pathways (the ubiquitin–proteasome system (UPS) and Ca^2+^) [2]. Autophagy is also an important regulator of the metabolism and homeostasis in many organs. Therefore, its involvement in skeletal muscle atrophy, cardiac remodeling, and the liver metabolism of cancer cachexia should be taken into consideration.

Studies have shown the complicated and changing role of autophagy in cancer [8,9,10]. In the early stage of cancer, autophagy acts as a tumor suppressor; thus, increasing autophagy could prevent cancer initiation [8]. However, in the advanced stages of cancer, autophagy is used by cancer cells to improve their fitness; therefore, inhibiting autophagy in cancer cells might serve as an option to improve cancer therapy [10]. As such, understanding the phase of cancer is important for recognizing the importance of autophagy activity. For cancer cachexia, which more prominently occurs in the later stages of cancer, the disbalance of autophagy causes uncontrolled cytokine production and secretion by inflammatory cells in the tumor and other organs [5]. Until recently, it has been debated whether using an autophagy modulator for cancer therapy is a wise decision, considering that autophagy also plays an important role in physiological functioning to maintain homeostasis [10].

Exercise, which is also well-known to modulate autophagy, is one of the best treatment options to support the recovery of patients with cancer cachexia [11]. The American College of Sports Medicine recommends 150 min (three to five days a week) of moderate-intensity aerobic exercise, two to three days a week of resistance exercise, and daily stretching for cancer survivors [12]. Preclinical studies in rodents concluded that exercise does not affect cancer cachexia, but could reduce the severity of cachexia in the later stages of cancer [13]. Human studies, on the other hand, have shown improvement of body weight and muscle mass in cachexia patients with lung and pancreatic cancer [14]. A combined exercise program might be a better choice for improving muscle wasting in cancer cachexia [15]. Recent studies have sought to delve deeper into the molecular mechanisms that occur in exercise before and after the development of cancer cachexia, especially those related to autophagy [16,17,18,19,20,21,22,23,24]. Therefore, understanding autophagy modulation in the skeletal muscle, cardiac muscle, and liver after exercise in cancer cachexia is of the utmost importance for suggesting exercise as an autophagy modulator in different stages of cancer.

## 2. Changes of Autophagy in Organs Related to Cancer Cachexia

**Figure 1:**
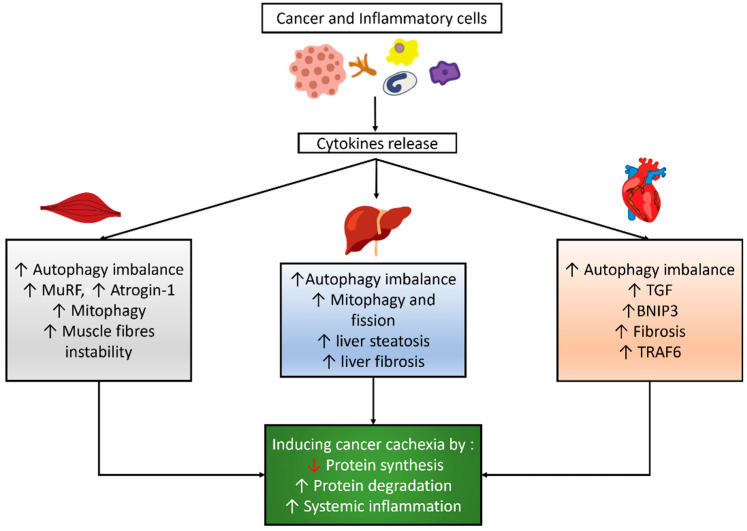
Autophagy modulation and its correlated effects in cancer cachexia. Cancer and inflammatory cells induce cytokines release which then results in an imbalance of autophagy and other effects in skeletal muscle, cardiac muscle, and the liver, which eventually lead to cancer cachexia.

Autophagy is a physiological intracellular process that consists of the destruction and elimination of substances, such as misfolded proteins or organelles, to adapt or maintain cellular homeostasis. This process is mediated by an autophagosome, which comprises a double-layer vesicle and undergoes fusion of the autophagosome to the lysosome [25]. Autophagy is a mandatory mechanism to maintain cell survival [26,27]. Many factors can induce autophagy, such as fasting, exercise, aging, disuse, and cancer [28,29,30,31,32]. Cancer and inflammatory cells might induce cytokine release, which influences the autophagy balance, mitophagy, and other signaling pathways in skeletal and cardiac muscles and the liver, as shown in Figure 1.

### 2.1. Autophagy Modulation in the Skeletal Muscle of Cancer Cachexia

Two main pathways induce protein degradation in cancer cachexia: UPS and autophagy [2]. UPS requires enzymes called E1, E2, and E3 to be activated and docked to a protein that will be degraded. E3 binds to a specific protein that soon will be docked to ubiquitin and transported to the proteasome. Two kinds of E3 have been recognized to be involved in muscle atrophy: MuRF1 and Atrogin-1. The skeletal muscles in cancer cachexia also expressed MuRF1 and Atrogin-1, which activate E3 enzymes, thus stimulating UPS pathways [33]. Autophagy also occurs through the process of engulfing ubiquitinated proteins via phagosomes and fusing them with a lysosome [34].

Hentilä et al. reported higher levels of lipidated LC3, LC3II/LC3I ratio, LC3b mRNA, and Beclin-1 in the skeletal muscle of C26 tumor-bearing mice. These findings were followed by increased p62 protein and unchanged p62 mRNA levels, suggesting a reduction in autophagosome clearance. These findings imply that autophagy, in conjunction with the UPS, leads to muscle atrophy [35]. Penna et al. measured the level of autophagy in the gastrocnemius muscle of C26 tumor-bearing mice and found that the levels of microtubule-associated protein 1 light chain 3B isoform I (LC3BI) remained unchanged, although the lipidated form (LC3BII) dramatically increased. Beclin-1, a key upstream regulator of autophagic sequestration, was significantly higher, implying that autophagy activation is a key to the early event in tumor-induced muscle depletion; p62 accumulated in a similar manner to Beclin-1 and LC3BII, which could indicate either the induction of autophagic sequestration or reduced autophagosome clearance [32].

### 2.2. Autophagy Modulation in the Cardiac Muscle of Cancer Cachexia 

In addition to skeletal muscle atrophy, cardiac remodeling is also induced by cancer cachexia, causing a condition known as cardiac cachexia [34,36]. Cancer cachexia might also induce cardiac remodeling that might disturb cardiac function, which is characterized by microscopic changes displaying cardiac atrophy because of the progressive loss of cardiac muscles [36,37].

The molecular mechanism underlying cardiac muscle atrophy is slightly different from the one that induces skeletal muscle atrophy in cancer cachexia [34,38]. Some studies showed an increase of UPS in the cachectic heart, although another study demonstrated that UPS did not increase, while autophagy did [38,39,40,41]. Cancer-induced cachexia implies a nutrient imbalance and cytokine production that affect cardiac muscle gene expression, causing an imbalance in cellular metabolism, inflammation, and necrosis [42]. In such conditions, cardiac muscles express transforming growth factor (TGF) and BNIP3, which induce autophagy, and then necrosis and fibrosis development [20]. Other markers, such as TRAF6, which is involved in apoptosis, and Beclin-1, which induces autophagy, are increased in cardiac muscle undergoing remodeling [34].

Cosper and Leinwand proved that the proteins of cathepsin L, Beclin, and LC3 were increased during cardiomyocyte atrophy in cancer, suggesting increased autophagy. They found a two-fold increase of cathepsin L mRNA in both male and female atrophic hearts, a 1.5-fold increase of LC3 mRNA, and a seven-fold increase of LC3II protein levels in male atrophic hearts, but only a three-fold increase in females [39]. Another study found a decrease of cardiac mass in the ApcMin/+ mouse model of colorectal cancer, together with an increase of Beclin-1 protein level, without a change of ubiquitination or apoptosis protein in the cardiac tissue. This result showed an increased autophagy process in cardiac cachexia, with no change of UPS stimulation and apoptosis [43].

### 2.3. Autophagy Modulation in the Liver of Cancer Cachexia 

Liver metabolism is also affected by cachexia via insufficient energy expenditure, phosphorylation oxidation, and lipid metabolism. The liver in cancer cachexia undergoes energy wasting by employing metabolic futile cycles that dissipate energy without anabolic or catabolic function [44]. Tumor cells induce the liver to produce energy via gluconeogenesis of lactate that is derived. The lactate is produced by tumor cells through aerobic glycolysis, known as the “Warburg effect” [45,46]. The liver also releases acute-phase proteins that alleviate inflammation and increase muscle protein breakdown [47,48]. Amino acids produced by skeletal muscle protein degradation provide further gluconeogenesis in the liver [44]. Energy wasting in cancer cachexia also decreases the phosphorylation oxidation capacity in mitochondria, which is marked by an increase of cardiolipin mediated by TNFα [49,50]. Recent research also showed that mitochondrial quality control (mitophagy and fission) was altered in cancer cachexia, in line with the development of hepatic fibrosis [51]. The liver cells in patients with cachexia have a decreased capability to distribute fat via very low-density lipoprotein (VLDL), along with an inability to oxidize fat production and deposition in liver cells, thus leading to liver steatosis [48]. These changes in liver metabolism are correlated with autophagy function, especially lipid droplet clearance, damaged mitochondria, protein aggregate removal, and liver fibrosis prevention [52]. Previous research strongly demonstrated that autophagy is critical to liver function, with lower autophagy linked to poorer results [53,54,55].

Rosa-Caldwell et al. showed that there was no link between the Beclin mRNA content and cancer progression. It was also observed that the LC3II/LC3I ratio, which acted as a surrogate measure of autophagosome production, showed a quadratic connection with cancer progression, with a 40–50% decrease in the LC3II/LC3I ratio at weeks one, two, and three, subsequently increasing at week four. The level of p62 mRNA did not change between the cancer groups, whereas the level of p62 protein increased over time as the disease progressed. In addition, it was also found that mitophagy and fission are altered before hepatic fibrosis occurs [51]. Another study found that the level of LC3II and the LC3II/LC3I ratio, as well as Beclin-1, increased in the liver of C26 tumor-bearing animals. In contrast to the skeletal muscle, an animal model of C26 colon cancer only showed elevated LC3I in the liver, with no change in p62, suggesting increased autophagosome formation without increased or decreased autophagic flux [35]. 

## 3. Exercise Modulates Autophagy in Cancer Cachexia

Exercise can increase muscle fibers through several mechanisms, such as mTOR, AMP-activated protein kinase (AMPK), and autophagy. [15] Exercise increases AMPK and triggers mTOR activity, increasing protein production, and thus resulting in a spike of protein quantities. MuRF1 and Atrogin-1 are suppressed by exercise, restoring protein degradation to its basal concentration, and thus decreasing muscle fiber breakdown. These mechanisms are correlated with each other, in which autophagy also has a crucial role. Autophagy is restored by exercise into a physiological condition, causing decreased muscle fiber degradation; however, it still maintains its function in degrading substances that may have a detrimental effect on cells [16].

Patients suffering from cachexia have engaged in different types and intensities of exercise. Studies on exercise intensity proved that high-intensity interval training was better than low- to moderate-intensity exercise, because it took a shorter time to achieve the expected results and patients endured less fatigue after exercise [56,57,58,59]. Aerobic and resistance exercises also have different benefits. Aerobic exercise is better at increasing capillarization, whereas resistance exercise is better at increasing the number of muscle fibers [60]. In the case of duration, a longer duration of exercise before cancer initiation provides better prevention of cancer cachexia than a shorter one [13]. These types and intensities of exercise can also be implemented in conjunction with chemotherapy [57,60].

The detailed mechanism for the correlation between the intensity, type, duration of exercise, and autophagy modulation in cancer cachexia remains far from being understood. In this review, we explored nine studies on the role of autophagy modulated by exercise in cancer cachexia. Eight studies were performed on animals [16,17,18,19,20,21,23,61], and one study was performed on humans [22], as summarized in Table 1. 

### 3.1. Aerobic Exercise Modulates Autophagy in the Skeletal Muscle of Cancer Cachexia Animals 

Endurance exercise has been proven to induce autophagy modulation in healthy skeletal muscles [62,63,64], but studies in cancer cachexia are still limited. Pigna et al. tailored a study using seven-week-old BALB/c female mice, which experienced cachexia induced by colon cancer and then died 19 days after. The study proved that aerobic exercise, AMP analog 5-aminoimidazole-4-carboxamide-1-beta-D-ribofuranoside (AICAR), and AMPK as “exercise mimetics” modulated autophagy in cancer cachexia induced by colon cancer. Voluntary wheel running in this study had the capability of restoring autophagy to its basal level, thus maintaining muscle homeostasis that eventually recovered muscle mass and function. A restored autophagy level was found in the tibialis anterior muscles of mice with colon cancer after voluntary wheel running, demonstrated by a reduction of the LC3II/LC3I and p62/GAPDH protein ratio. In healthy muscle, there is a balance in the physiological autophagic system between LC3 (autophagosome production) and p62 (clearance) that prevents muscle wasting. A dysregulated autophagic system is found in the C26 muscle, demonstrated by increased LC3 and p62 that might be correlated with muscle wasting. As a potent inducer of autophagy, exercise restores the physiological autophagic system by balancing the production and clearance, resulting in preserved muscle mass in cancer cachexia. Exercise was shown to rescue the muscle mass, fiber size, morphology of the basement membrane, fatigue time, and even the distance of voluntary running, correlated with the life span of cancer mice [16].

The study also investigated the potential therapeutic use of AICAR as an AMPK activator that might induce autophagy by inhibiting mTOR while activating FoxO3A and rapamycin as a specific inhibitor of mTOR; thus, these two drugs mimic the effects of exercise [65,66,67,68]. AICAR and rapamycin were given by peritoneal injection to two groups, one of C26 tumor-bearing mice and one of controls, and the result showed that the two drugs prevented skeletal muscle wasting and reduction of body weight in C26 tumor-bearing mice. The role of autophagy in this process was ruled out by the finding of increased LC3II/LC3I and p62/GAPDH protein levels, as well as an in vitro study using C2C12 myotubes treated with C26 cells and chloroquine, which showed worsening muscular atrophy as a result of inhibited autophagy [16].

It was concluded that autophagy directly induced atrophy of the skeletal muscle in cancer cachexia [16], a finding that was supported by other studies that claimed that increased autophagy is correlated with muscular atrophy and the deleterious effect of blocking autophagy in cancer cachexia [32,69]. Voluntary wheel running and treatments with AICAR and rapamycin were suggested to counteract cachexia by correcting the previously disturbed autophagic flux [16].

Morinaga et al. designed a study using 10–11-week-old female wild-type BALB/c mice injected with C26 colon cancer. Treadmill running was performed 30 min/day, five days/week, 12 m/min, until four weeks after tumor injection. Aerobic exercise increased the cross-sectional area and improved muscle atrophy in the tibialis anterior and gastrocnemius muscles; however, it failed to decrease weight reduction in the soleus muscle. The difference in effect might be influenced by the dominant fibers in each skeletal muscle. Protein synthesis signaling (p-Akt, p-mTOR, p70S6, and 4EBP-1) and adiponectin-related genes (adiponectin, AdioR1, and APPL1) were found to have significantly increased after aerobic exercise; meanwhile, LC3II was not significantly decreased, suggesting that aerobic exercise might not be sufficient to restore autophagy to the basal level in the early stage of cachexia. Adiponectin is a hormone produced by adipose tissue that plays important roles in lipid metabolism, vascular remodeling, and insulin sensitivity [21,70,71]. Research confirmed the role of adiponectin in cancer cachexia through an in vitro study by administering recombinant adiponectin on C2C12 myotubes with an added C26-conditioned medium. This recombinant adiponectin treatment resulted in increased p-Akt, p-mTOR, and p70S6, and decreased LC3II expression. It was suggested that adiponectin plays a potential role in decreasing autophagy by activating mTOR. Pigna et al. and Morinaga et al. showed a slight difference in autophagy modulation by aerobic exercise depending on the exercise protocols and cachexia stage [21].

### 3.2. Resistance Exercise Training Modulates Autophagy in the Skeletal Muscle of Cancer Cachexia Animals 

As a well-known type of exercise that stimulates muscle hypertrophy, resistance training is predicted to be more beneficial than aerobic exercise for counteracting cachexia; however, further investigation is needed to support this hypothesis [13]. Resistance training is proven to upregulate the phosphorylation of the Akt-mTOR/p70 pathway [72,73] and synthesis of myofibrillar protein, thus increasing the contractility property of skeletal muscle [74,75]. One study compared aerobic and resistance training in cancer cachexia, but found no difference in the effect for preventing body mass reduction or improving the skeletal muscle fiber area in the C26 mouse model of cancer cachexia, despite its effect in increasing the mRNA levels of IGF-1 and myogenin [76].

Neves et al. designed a short-term resistance exercise training (RET) plan, using a canvas jacket to fix rats to the apparatus while performing a “squat-like movement” with electrical stimulation. The intensity was a 65% maximum lifted weight and three sets of 10 repetitions in the period of the “survival window” (±18 days). The overload was decreased 20% during tumor progression if the animal was incapable of finishing the RET set. The tumor was induced by injecting Walker 256 cells into the bone marrow of male Wistar rats, and the plantaris and extensor digitorum longus (EDL) muscles’ atrophy was detected 15 days after the injection. The study concluded that RET was not effective in improving skeletal muscle wasting caused by cancer cachexia. Atrogin-1 and MuRF1 protein levels were unchanged between the control and tumor groups, but the p62 protein and LC3II/LC3I ratio were increased in tumor groups compared with control and exercise groups. These effects suggest an imbalance or dysregulation of autophagy during cachexia progression. For intensity, this study failed to perform a high-intensity RET protocol on tumor-bearing rats. Nevertheless, this study succeeded in confirming that the mortality of rats with cancer cachexia was associated with the loss of strength capacity [23].

### 3.3. Combined Exercise Modulates Autophagy in the Skeletal Muscle of Cancer Cachexia Animals 

Considering the different effects of various exercise types on cancer cachexia, a combination of different types of exercise could provide a better result for counteracting cancer cachexia. Combined exercise downregulates inflammation, improves body composition, and increases the strength of skeletal muscle, more than resistance or aerobic exercise alone [18,77,78,79,80].

Ranjbar et al. investigated the effect of combined exercise (resistance and endurance exercises) on tumor-bearing male BALB/c mice that had been inoculated with C26 carcinoma cells. The combined exercise consisted of aerobic and resistance exercise sessions, conducted from four weeks before until 11 days after tumor inoculation. Resistance exercise in the study was conducted using a one-meter ladder and 85° inclination, with three sets of two repetitions without electrical stimulation. A motorized wheel was used in the aerobic exercise, 3 × 10 min per day, with five meters/min. The results showed a decreased LC3BII/LC3B1I ratio of protein levels and an increased mRNA relative expression of LC3B in the tibialis muscles of combined exercised C26 tumor-bearing mice compared with C26 hosts. The research showed that combined exercise was effective in modulating the sequestration of autophagy. As for the p62 levels, there was no change between sedentary C26 hosts and combined exercised C26 tumor-bearing mice, and the protein levels and mRNA relative expression of p62 remained high in both groups. However, the changes of Atrogin-1 and MuRF1 mRNA relative expression found in gastrocnemius muscles were only marginal; the researchers only found a reduction that was close to significant between C26 hosts and combined exercised C26 tumor-bearing mice [18].

Another factor contributing to cancer cachexia is an impaired redox balance that might influence mitochondrial function [17,81]. A low level of reactive oxygen species (ROS) is needed to maintain skeletal muscle function and homeostasis, but a high level of ROS induces cellular damage that leads to dysfunction and disrupted homeostasis in the skeletal muscle [82,83]. Increased ROS was found in animals with cancer cachexia, which proved to be correlated with protein degradation in the skeletal muscle [84,85,86]. Studies have shown that ROS induces autophagy and that autophagy delivers negative feedback for ROS production [83]. After ROS is produced in mitochondria and by Nox isoform 2, it activates or inhibits autophagy through PIK3K/Akt/mTORC1, p38/p53, AMPK, and FOXO3-BNIP [83,87,88,89,90]. Autophagy impairment in cancer cachexia induces protein accumulation that promotes mitochondrial damage and finally increases ROS production even more [83,91].

The effects of exercise on restoring the redox balance depend on the type, intensity, and timing of the exercise [17]. Moderate-intensity exercise induces metabolic adaptation in correlation with antioxidant capacity, thus decreasing systemic inflammation [17,92,93]. Ballaro et al. designed a study using male BALB/c mice injected with C26 colon carcinoma cells and engaged in combined moderate exercise using a custom motorized wheel with 11 m/min speed, uphill, for 45 min a day, three days out of four, until 12 days after tumor inoculation. They studied the effects of exercise on relieving muscle wasting and function and on the redox imbalance, autophagy, mitochondrial mass, and mitophagy. The results showed a decrease in LC3BII, the LC3II/LC3I ratio, and p62 protein levels in the gastrocnemius muscles of exercised C26 tumor-bearing mice, although the p62 protein level did not significantly differ from that in C26 hosts; this might be caused by the high variability intragroup. The same pattern was also observed for Map1lc3b, sqtm1, and Ctsl1 mRNA’s relative expressions in the tibialis muscles of exercised C26 tumor-bearing mice; however, no effects for the Lamp2 and Beclin-1 mRNA’s relative expressions were found. An increase of the Beclin-1 protein level in C26 hosts was observed when compared with the sedentary controls, although there was no difference when compared with the exercised C26 tumor-bearing mice [17]. The study also found that ROS levels were reduced, the Nrf2/Keap1 ratio steadily unchanged, and GSH levels altered in exercised C26 tumor-bearing mice compared with controls, suggesting that moderate exercise only partially restored the redox and autophagy dysregulation [17].

Oxidative stress damaged mitochondria, the degradation of which was mediated by mitophagy [94]. Increased PINK1, as a mitophagy marker in C26 hosts, confirmed the increased level of mitophagy in cancer cachexia, although exercise did not change it. BNIP3, another mitophagy marker, was found upregulated only in exercised healthy mice, although there were no differences in C26 tumor-hosts and exercised C26 tumor-bearing mice when compared with sedentary controls [17]. The degradation process of mitochondria must be followed by mitochondrial biogenesis to achieve mitochondrial homeostasis [95]. Mitochondrial biogenesis and mass were found to be increased by exercise in C26 tumor-bearing mice, confirmed by increases of the PGC1α and cytochrome c protein levels, which suggested effective mitochondrial turnover [17].

### 3.4. Exercise Modulates Autophagy in the Skeletal Muscle of Chemotherapy-Induced Muscle Wasting 

As one of the anticancer treatments, chemotherapy is correlated with well-known side effects such as nausea, vomiting, fatigue, and anorexia. Its molecular mechanism increases protein catabolism, damages the mitochondria, and induces skeletal muscle wasting [96,97,98]. Autophagy modulation by exercise in cancer cachexia patients treated with chemotherapy should be considered as a means to improve their quality of life. Ballaro et al. designed exercises that were mentioned previously in this paper to compare the effect of 28 days of chemotherapy with or without combined moderate exercise after C26 tumor injection in female and male six-week-old BALB/c mice. The chemotherapy regimens used were oxaliplatin and 5-fluorouracil (oxfu), with protocols based on previous studies that prolonged the survival rate in an animal model [99,100]. Exercise improved muscle wasting in C26 tumor-bearing and C26 oxfu mice; meanwhile, at six to eight weeks after tumor inoculation (late phase of cancer cachexia), exercise intolerance correlated with a shorter survival rate was provoked. Exercise also decreased proteolysis, which was confirmed by decreased autophagy (Beclin-1, LC3BI, and LC3BII gene expression, and p62 protein level) without an increase in protein synthesis (p-Akt, S6, p-AMPK) in C26 tumor-bearing and C26 oxfu mice. A further change in mitochondrial homeostasis was also investigated, and exercise was found to improve the mitochondrial mass (PGC-1α, cytochrome c, SDHA protein levels, SDH activity, and ATP content) and decrease mitophagy (BNIP3 and PINK1 protein levels and Park2 gene expression) in C26 oxfu mice. Improved mitochondrial function was also supported by an increase in Mfn2 gene expression indicating fusion, with no change in Fis1 and Mfn1 gene expression indicating fission, in exercised C26 oxfu mice [61].

Another study in patients with different types of cancer treated with chemotherapy attempted to investigate the molecular signaling pathways behind combined exercise to prevent muscle wasting. Ten female patients with cancer in comparison with ten healthy subjects were biopsied three times. The exercise protocol consisted of aerobic and resistance exercises (knee extension, leg press, etc.) and an aerobic exercise (ergometer bicycles) with progressively increased intensities. The results showed an increase of type II muscle fibers, but no change in type I muscle fibers, found together with increased strength of the knee and elbow extensor muscles. Exercise increased metabolism (GLUT4); however, there was no change in mitochondrial proteins (p-AMPK, Cyt-C, COX-IV, PDH, SDHA, and VDAC). Autophagy (LC3BII/LC3BI, ATG5, ULK1), the UPS system (FOXO3, MURF1, ATROGIN-1), and protein synthesis (mTOR, 3EBP1, S6rp) were dysregulated during chemotherapy, but exercise leveled off their levels, proving its potential benefit in preventing further disruption by chemotherapy and cancer cachexia [22].

### 3.5. Exercise Modulates Autophagy in the Cardiac Muscle of Cancer Cachexia Animals 

Cancer cachexia induces wasting not only in the skeletal muscle but also in the cardiac muscle because cardiac remodeling leads to cardiac dysfunction [34]. Cardiac wasting occurs because of cytokines released by the tumor, the release of danger-associated molecular patterns, decreased insulin signaling, and autophagy [34,101,102,103]. Cardiac cachexia might be prevented by modulating those mechanisms. Aerobic exercise training has been proposed as a part of strategic plans against cardiac cachexia, for prevention and treatment [104]. Limited studies have proven the benefits of aerobic exercise for treating cardiac cachexia in cancer animal models [19,20,105,106,107].

Using CT26 (colon adenocarcinoma) injected in the right flank of male BALB/c mice, Fernandes et al. explored the impact of aerobic exercise with moderate intensity on tumor-bearing mice. Aerobic exercise was performed for 60 min per day, five times a week, at a 60% maximum speed, from 30 days before until 15 days after tumor injection. Cardiac cachexia was found in CT26 tumor-bearing mice, which was confirmed by the decrease of cardiomyocyte diameter, left ventricle ejection fraction, necrosis, and fibrosis that was attenuated by aerobic exercise. Fibrosis attenuation was shown by a decrease in TGF-β1 mRNA levels in exercised CT26 tumor-bearing mice. As for autophagy, the study found an upregulation of ATG7, BNIP3, and LAMP2 mRNA levels in the cardiac tissues of CT26 mice [19]. BNIP3 plays the main role in regulating autophagy, mitochondrial function, and necrosis, especially in cardiac cells [19,108,109]. After 45 days of moderate treadmill exercise, there was a reduction in the mRNA levels of BNIP3, which supported the hypothesis that aerobic exercise training might be beneficial in reducing the cardiac remodeling of cancer cachexia by modulating autophagy [19]. The study also found restoration of mitochondrial complex II and IV after aerobic exercise, which proved its benefits for mitochondrial homeostasis, leading to better control of protein quality in heart failure that may evoke in cardiac cachexia [19,110].

Parry and Hayward used mammary adenocarcinoma MAT-B-III to induce cardiac cachexia in female Fischer 344 rats. Aerobic exercise in the form of voluntary wheel running was performed four weeks before until two weeks after tumor injection. At the end of the study, sedentary tumor-bearing rats displayed cardiac atrophy and impairment of cardiac function, accompanied by a shift from αMHC to βMHC and increased autophagy flux (an increase of LC3II and a decrease of p62 protein levels). Exercised tumor-bearing rats showed no cardiac atrophy, preserved cardiac function, decreased shifting from αMHC to βMHC, and reduced LC3BII with no change of p62 protein levels, which emphasized a decrease in autophagosome formation that might be correlated with autophagy attenuation [20]. The upregulation of autophagy under specific conditions, such as cardiac ischemia, has a positive impact on cardiac function, but maladaptive autophagy in cancer can induce fibrosis and cardiac dysfunction that eventually lead to heart failure [20,111,112]. Considering the different effects of exercise type on cancer cachexia, a combination of different types of exercise could provide a better result for counteracting cancer cachexia. Combined exercise downregulates inflammation, improves body composition, and increases the strength of skeletal muscle, more than resistance or aerobic exercise alone [18,77,78,79,80].

### 3.6. Exercise Modulates Autophagy and Potentially Influences Liver Metabolism in Cancer Cachexia 

As cancer cachexia induces muscle wasting in the skeletal and cardiac muscles, it induces energy-wasting in the liver. A study by Donatto et al. showed that eight weeks of RET improved the plasma profile, reduced steatosis by increasing liver fat oxidation, decreased the TNF-α/IL-10 ratio, and increased the levels of anti-inflammatory myokines such as IL-6 and IL-10 [113]. Endurance training also showed similar results in the liver and adipose tissue [113,114,115,116]. For intensity, moderate and high intensities are suggested to modulate liver metabolism [114,115,116,117]. The study by Guarino et al. proved that exercise could increase autophagy (increase of LC3BII/LC3BI ratio and PINK1 and tendency toward mTOR and ATG5 downregulation) in the liver. They also found that impeded tumorigenesis leads to hepatocellular carcinoma, as confirmed by a decrease in liver nodules found after exercise plus NASH (Nonalcoholic Steatohepatitis) [118]. Nevertheless, autophagy modulation after exercise in the liver of cancer cachexia remains unclear. Further investigation might reveal the best type, intensity, and duration of exercise to cope with dysregulation of lipid metabolism that might induce liver steatosis in cancer cachexia through autophagy modulation.

## 4. Conclusions

**Figure 2:**
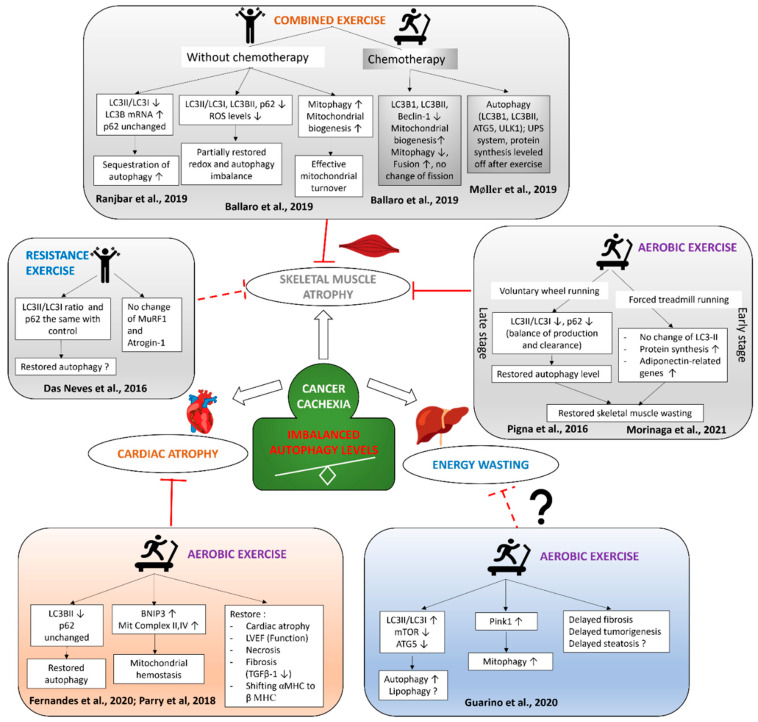
Exercise modulates autophagy in the skeletal and cardiac muscles and liver of cancer cachexia.

Exercise modulates autophagy in skeletal and cardiac muscles and its potential to influence liver metabolism in cancer cachexia is presented in Figure 2. Careful evaluation and personalization before choosing the type, intensity, and duration of exercise to cope with cancer cachexia might leverage the benefits of exercise. Research into the molecular mechanism of autophagy modulation by exercise based on different exercise types has shown that combined or aerobic exercise alone is better at restoring the disbalance of production and the clearance of autophagy, which is believed to induce muscle wasting. Restoring autophagy was also correlated with increased anti-inflammatory responses and mitochondrial dynamics related to mitophagy; it prevented further disruption by chemotherapy, improved cardiac cachexia, and could potentially interfere with steatosis prevention and energy-wasting in the liver. Further studies into the molecular mechanism behind autophagy modulation after exercise in cancer cachexia are still needed. Using exercise mimetics for those who experience exercise intolerance might be considered as an alternative for treating cancer cachexia. In conclusion, “tailor-made” exercise for each patient experiencing cancer progression seems to be the best option to counteract cancer cachexia in the future.

## Figures and Tables

**Table 1:** Summary of autophagy modulation by exercise in cancer cachexia.

No	Study	Model	Tumor Type/Induction Model	Exercise Modality	Exercise Prescription	Exercise Initia tion	Results	Reference	Organs
1	Pigna et al., 2016	Seven-week-old BALB/c female mice	Colon adenocarcinoma cells CT26	Voluntary running	Ad lib voluntary wheel running	Since day one of implantation in tumor-bearing mice	Restored atrogin1, murf1, LC3, and p62 in tibialis anterior muscle to its physiological level	[16]	Tibialis anterior muscle
2	Morinaga et al., 2021	Female 10–11 week old, wild type BALB/c mice	Colon carcinoma cells C26	Treadmill exercise	Treadmill running 12 m/min, 20 min/day, 5 days/week.	Treadmill habituation 4–5 days, continued with exercise from the day after injection until four weeks after injection	No change of LC3-II protein synthesis (pAkt, pmTOR, p70S6, and 4EBP-1), and adiponectin-related genes (adiponectin, AdioR1, and APPL1) increased.	[21]	Tibialis anterior, gastrocnemius, and soleus muscles
3	Das Neves et al., 2016	14-week-old Wistar rats	Walker 256 tumor cells in the bone marrow	Resistance exercise training	Eight sessions of RET (each session included three sets of repetition at the intensity of 65% maximum lifted weight (1-RM)	11 days before tumor injection until 15 days post tumor injection	Increased p62 protein content and LC3 II/I ratio were found in EDL muscles of the tumor groups when compared to the control and exercise groups.	[23]	EDL (extensor digitorum longus) muscle
4	Ranjbar et al., 2019	Six-week-old male BALB/c mice	Colon cancer cells C26	Combined (resistance and endurance training)	Resistance: climbing a 1-m ladder with 1.5-cm grid steps and inclined 85°, aerobic using a motorized wheel 25 min from 5–9 m/min	Four weeks before tumor implantation until 11 days after injection of C26 cells	Decreased LC3BII/I ratio in exercise C26 tumor-bearing mice compared to C26 hosts, p62 steadily increased in C26 tumor hosts and exercised C26 tumor-bearing mice	[18]	Gastrocnemius muscle and tumor
5	Ballaro et al., 2019	Six-week-old male BALB/c mice	Colon cancer cells C26	Treadmill exercise (mix resistance and endurance training)	Custom motorized wheel (radius = 16 cm), 45 min a day, moderate speed (11 m/min)	Adapted to motorized wheel five days before implantation, exercise three days out of four, until 12 days after injection of C26 cells	Decreased LC3BII/I ratio, p62 protein levels in gastrocnemius muscles, and Map1LC3B, sqtm1, ctsl1, and mRNA relative expression in tibialis muscles of exercised C26 tumor-bearing mice compared to C26 hosts.	[17]	Gastrocnemius and tibialis muscles
6	Møller et al., 2019	10 cancer patients	Seven breast cancer patients, one head and neck cancer, one rectal cancer, one sarcoma cancer patient	Combined (resistance and aerobic exercise)	Three 90-min sessions per week: six resistance exercises (knee extension, leg press, lateral pull-down, chest press, back extension, and sit-ups); aerobic: ergometer bicycles	4–7 weeks after chemotherapy until 10 weeks after a combination of chemotherapy and exercise	No change of LC3BII/I ratio, p62, ULK1, and ATG5 in vastus lateralis between the combination of exercise and chemotherapy alone, but they all leveled off after exercise	[22]	Vastus lateralis muscle
7	Ballaro et al., 2019	Female and male six-week-old BALB/c mice	Colon carcinoma cells C26	Treadmill exercise (mix resistance and endurance training)	Custom motorized wheel (radius = 16 cm), 45 min a day, moderate speed (11 m/min)	Adapted to motorized wheel five days before implantation, then: exercise three days out of four until 12 days after injection of C26 cells; exercise two days, rest one day (chemo); and three days, rest one day, until 28 days after injection	Decrease autophagy (Beclin-1, LC3BI, and II, p62) in exercised C26-bearing and C26 oxfu mice. Improve mitochondrial mass (PGC-1α, cytochrome c, and SDHA, SDH activity, ATP content), decrease mitophagy (BNIP3 and PINK1, Park2) in exercised C26 oxfu mice. Increase fusion (Mfn2), no change of Fis1 and Mfn1 (fission)	[61]	Gastrocnemius muscle
8	Fernandes, 2020	6–12-week-old male BALB/c mice	Colon adenocarcinoma cells CT26	Treadmill running	Moderate AET 60 min per day, five times/week	30 days before + 15 days after injection	Increased BNIP3, LAMP2, and ATG12 in CT26 mice, reduced BNIP in cardiac muscles of CT26 exercise tumor-bearing mice	[19]	Cardiac
9	Parry and Hayward, 2018	Twelve-week-old female Fischer 344 rats	A rat mammary gland tumor cell line, 13762 MatBIII	Voluntary running	Ad lib voluntary wheel running	Four weeks before until two weeks after tumor implantation	Reduction of LC3II protein level in wheel running tumor-bearing rats compared to sedentary tumor-bearing rats, but no change in p62 protein levels	[20]	Cardiac

## Data Availability

Not applicable.
